# HIV-1 Tat Forms a Dissociable Complex with 7SK snRNP and Exhibits Clade Differences in Reversing the Sequestration of P-TEFb

**DOI:** 10.3390/v18101137

**Published:** 2026-10-14

**Authors:** Amie Donner, Taebin Son, Sanjna Boda, Claire T. Kenney, Alexis Chiandussi, Rania Rodríguez, Jonathan Karn, Uri Mbonye

**Affiliations:** 1 Department of Molecular Biology & Microbiology, Case Western Reserve University School of Medicine, Cleveland, OH 44106, USA; 2 Department of Biology, University of Puerto Rico, Mayaguez 00681, Puerto Rico

**Keywords:** HIV Tat, P-TEFb, 7SK snRNP, HIV latency, HIV clades, transcription elongation, latency reversal, virus–host interactions

## Abstract

Recent efforts to employ ectopic Tat as a latency-reversal strategy can benefit from the identification of Tat variants with more efficient transactivation activity. Tat primarily serves as a recruiter of the host transcription elongation factor P-TEFb to proviral HIV to stimulate transcription elongation. Tat can also functionally interact with a host of chromatin-modifying enzymes to promote HIV transcription initiation. In actively dividing T cells, P-TEFb is kept inactive by the 7SK snRNP complex. Although Tat is capable of physically extracting P-TEFb from 7SK snRNP as well as forming an assembly with the sequestrator complex, it is unclear whether Tat:7SK snRNP (Tat:7SK) is a reversible functional intermediate that mediates the recruitment of P-TEFb to proviral HIV. This study explored the reversibility of Tat:7SK and whether Tat clade variants differ in their ability to assemble this complex, mobilize P-TEFb and transactivate latent HIV. While the ectopic expression of clade B NL4-3 Tat caused a substantial dissociation of P-TEFb from 7SK snRNP, affinity tag-based immunoprecipitation experiments confirmed the assembly of Tat:7SK with the displacement of the P-TEFb inhibitory component HEXIM1. The P-TEFb kinase inhibitor flavopiridol (FVP), known to rapidly dissociate P-TEFb from 7SK snRNP, also induced the efficient release of Tat:P-TEFb from Tat:7SK, suggesting that P-TEFb remains a component of this latter complex. While Tat variants representing clades A, B, C and CRF_AE exhibited comparable binding to P-TEFb, clade D Tat showed the least binding. Unlike clades B and CRF_AE, clades A and C Tat associated much more weakly with the core 7SK snRNP subunits MEPCE and LARP7, indicating that their bound P-TEFb is likely liberated. Evaluation of latent HIV reactivation in the Jurkat 2D10 clonal model revealed a rapid loss of Tat transactivation capacity over time across all clades examined, with the most striking disparity observed between Clades A and D. Overall, our findings reveal substantial clade-specific differences in Tat’s ability to liberate P-TEFb from 7SK snRNP and transactivate latent HIV, with the clade A variant displaying the highest potency. Contrary to the prevailing paradigm that Tat:7SK is a “dead-end” complex that is unable to avail P-TEFb for transcriptional control, we show that it is a reversible intermediate from which Tat:P-TEFb can be released *en bloc*.

## 1. Introduction

HIV latency, established primarily within minor subsets of resting memory CD4^+^ T cells, is refractory to antiretroviral therapy (ART) and has therefore been identified as the definitive barrier to a cure [1,2]. The “Shock and Kill” strategy, involving the deliberate reactivation of latent HIV to trigger cell death or immune clearance, remains a research focus for eliminating the persistent reservoir or achieving ART-free remission. However, this approach has seen limited success, primarily because non-toxic latency-reversing agents (LRAs) that can effectively stimulate viral RNA synthesis remain to be discovered. The marginal effects of histone deacetylase inhibitors at reversing HIV latency—observed in both ex vivo primary cells and in several clinical trials [3,4,5,6,7,8,9,10]—has provided a clear indication that epigenetic silencing of the proviral promoter is but one of the major repressive mechanisms responsible for maintaining HIV in a transcriptionally inert state in memory CD4^+^ T cells. Mechanistic studies conducted using T cell models of HIV latency have shown that restriction of the activity of the host transcription elongation factor P-TEFb constitutes an additional block to the reactivation of transcriptionally latent HIV [11,12,13,14]. Among LRAs that can bypass epigenetic blocks and stimulate P-TEFb activity, the viral trans-activator protein Tat is considered ideal because it does not trigger global T cell activation in primary cells [15,16,17].

HIV Tat is essential for transitioning from viral latency to productive transcription by playing a dual role: it recruits P-TEFb to proviral HIV to drive transcription elongation and functionally interacts with chromatin-modifying enzymes to promote initiation [18,19,20,21,22,23,24,25,26,27,28,29,30,31,32,33,34]. Entry of promoter-proximally paused RNA polymerase II (RNAP II) into productive elongation at many protein-encoding genes requires the kinase activity of P-TEFb, a heterodimer complex of CDK9, the kinase subunit, and its regulatory partner cyclin T1 (CycT1) [22]. Tat and P-TEFb cooperatively bind TAR, a 59-nucleotide RNA hairpin structure formed shortly after initiation of HIV transcription, which places the enzyme in close proximity to inefficiently transcribing RNAP II complexes [35]. P-TEFb extensively phosphorylates the C-terminal domain (CTD) of the large subunit of the polymerase at Ser2 of the heptad repeats Y-S-P-T-S-P-S as well as a linker region between the polymerase core and CTD [36,37,38]. Additional phosphorylation by P-TEFb occurs on negative elongation factors responsible for reinforcing RNAP II pausing, namely NELF and DSIF [39,40]. The overall effect of these phospho-modifications by P-TEFb is to remove the blocks to elongation imposed by NELF and DSIF, and to stimulate efficient elongation and co-transcriptional processing of proviral transcripts.

In actively dividing cells, including transformed T cells, a significant portion of assembled P-TEFb (>50–90%) is found to be reversibly associated in the nucleus with the ribonucleoprotein (RNP) complex 7SK snRNP [41,42,43,44], that inhibits P-TEFb kinase activity and sequesters the enzyme in nucleoplasmic regions found in close proximity to genomic sites of active transcription [45,46]. Phosphorylation of CDK9 on its activation loop at Thr186 is required for its assembly with CycT1 and incorporation of the P-TEFb heterodimer into 7SK snRNP [42]. Central to this inhibitory complex is 7SK snRNA, a highly conserved 332-nucleotide RNA polymerase III transcript that provides a molecular scaffold to coordinate the binding of two molecules of P-TEFb to a homodimer of the inhibitory proteins HEXIM1 or HEXIM2 [47]. Binding of HEXIM1 to the 5′ hairpin of 7SK (Stem-Loop; SL1) is a prerequisite for the association of P-TEFb with the 3′ hairpin (Stem-Loop; SL4) [48,49]. Recent studies employing NMR and biophysical methods have identified two high affinity sites on SL1 of 7SK snRNA whose binding by an HEXIM1 homodimer induces a conformational switch in HEXIM1 that unmasks a PYNT motif and an acidic region which are essential for binding and inhibiting P-TEFb [49]. By employing tandem mass spectrometry, we previously identified the dual phosphorylation of HEXIM1 at Tyr271 and Tyr274 located immediately upstream of the C-terminal coiled-coil homodimerization domain [50]. Phosphomimetic substitution of these two tyrosine residues (Y271E/Y274E) disrupts the incorporation of P-TEFb into 7SK snRNP and causes a redistribution of CDK9 into the cytoplasm. These findings have led to the proposition that, in addition to inhibiting the kinase, 7SK snRNP functions to retain P-TEFb in the nucleus to provide an exchangeable pool of the enzyme that can be readily accessed to stimulate transcription.

While the dissociation of 7SK snRNP is partially responsive to extracellular stimuli of various signaling pathways as well as cellular stress induction [41,44,51,52,53], it is more profoundly sensitive to CDK9 kinase inhibitors [42,54]. The C-terminal P-TEFb-interacting domain of BRD4, a major recruiter of P-TEFb to cellular genes, is also known to be capable of physically dislodging P-TEFb from 7SK snRNP through as-of-yet unclear mechanisms [55]. To be able to recruit P-TEFb to proviral HIV effectively, Tat has evolved the molecular ability to outcompete BRD4 in binding and remodeling 7SK snRNP. Unlike BRD4, which extracts P-TEFb from 7SK snRNP in the absence of any known interactions with the RNA scaffold, Tat binds 7SK at SL1 analogously to TAR RNA to displace the HEXIM1 homodimer [56,57,58]. The first study to identify the assembly of Tat:P-TEFb:7SK snRNA (Tat:7SK) also described the complex as being stably resistant to dissociation by stimuli, stress signals or agents known to disrupt 7SK snRNP [59]. Thus, whether Tat:7SK is a bona fide functional intermediate essential for the redirection of P-TEFb to TAR RNA anchored at the proviral locus remains to be determined.

Here, we explored whether Tat forms a reversible intermediate with 7SK snRNP that mediates the recruitment of P-TEFb to proviral HIV. Fluorescence imaging revealed a strong colocalization of Tat and 7SK snRNA in the nucleoplasm of HIV-infected primary T cells, suggestive of Tat:7SK assembly in a physiologically relevant model. Contrary to the notion that Tat:7SK is a “dead-end” complex in which P-TEFb cannot be availed for transcriptional control, we demonstrate that it is a dissociable complex whose intracellular assembly can be rapidly reversed by a brief cell treatment with flavopiridol (FVP), a selective CDK9 kinase inhibitor. The dissociation of Tat:7SK by FVP observed using intact cells as well as with treated whole-cell extracts suggests that it is a direct result of inhibitor binding to CDK9. These observations ascertain the presence of P-TEFb in Tat:7SK and demonstrate that liberation of the enzyme is coupled to its interaction with Tat. Since geographically diverse HIV clades exhibit sequence variation in the known protein–protein interaction regions of Tat, this study also examined whether the trans-activation of latent HIV is influenced by clade differences in the ability of Tat to bind P-TEFb and assemble with 7SK snRNP in cells. Our findings demonstrate that Tat clade variants vary in their capacity for P-TEFb binding, Tat:7SK assembly, proviral reactivation, and cellular restriction, with clade A proving superior for HIV latency reversal.

## 2. Materials and Methods

### 2.1. Cell Line and Primary Cell Culture

Jurkat and HEK 293 cells expressing FLAG-Tat variants were cultured in complete RPMI 1640 medium (supplemented with 10% fetal bovine serum, 100 IU/mL penicillin, 100 µg/mL streptomycin, 100 µg/mL normocin, and 25 mM Hepes, pH 7.2). Memory CD4^+^ T cells that were used for immunofluorescence and RNA FISH imaging were isolated from commercially obtained and de-identified, healthy donor-derived peripheral blood mononuclear cells using the EasySep Memory CD4^+^ T cell Enrichment Kit (StemCell, Cambridge, MA, USA). Isolated memory cells were allowed to recover overnight in complete RPMI medium lacking IL-2, then either stimulated with T cell receptor (TCR) activator Dynabeads for 24 h or left unstimulated. Generation of the ex vivo primary Th17 cell model of HIV latency, known as the Quiescent Effector Cell Latency (QUECEL) model, has previously been described in extensive detail [60,61,62]. Latently infected primary Th17 cells were cultured in complete RPMI medium then either stimulated with TCR activator Dynabeads for 24 h or left unstimulated.

### 2.2. Recombinant Tat Expression Constructs

Representative Tat variants of Clades A (U455), B (full-length HXB2), C (92BR), D (Eli) and CRF_AE (CM240) were custom-synthesized as FLAG-tagged, codon-optimized gBlocks gene fragments (Integrated DNA Technologies, Coralville, IA, USA). BamHI and EcoRI flanking restriction sites were used to sub-clone these fragments onto the pMSCV puromycin-resistant expression vector (Takara Bio, San Jose, CA, USA). Successful generation of MSCV FLAG-Tat constructs was confirmed by sequencing the entire insert using the MSCV primer 5′-CCCTTGAACCTCCTCGTTCGACC-3′.

### 2.3. Transient Transfection of HEK 293 Cells

Adherent HEK 293 cells were cultured in T150 flasks to 80% confluency prior to replacing the cell culture media with 15 mL of fresh complete medium. Briefly, for each transfection, 10 µg of pMSCV FLAG-Tat construct was resuspended in 1.5 mL Opti-MEM medium then mixed with 54 µL 1 mg/mL polyethylenimine (PEI) at room temperature for 15 min. Cells were transiently transfected by addition of the DNA/PEI mixture and incubation for 24 h at 37 °C.

Single-round packaged MSCV virus for infecting Jurkat cells and generating stable cell lines were also prepared by co-transfecting a T150 flask of HEK 293 cells with a DNA/PEI mixture containing 7.5 µg of pMSCV FLAG-Tat, 7.5 µg of the pCMV-dR8.91 retroviral gag/pol vector, and 3 µg of pCMV-VSVG (Envelope VSVG). Cell culture medium containing packaged virus was harvested 48 h post-transfection and cleared by centrifugation at 450× *g* for 3 min as well as by passing the supernatant through a 0.22 µm vacuum-driven filter (Millipore-Sigma, St. Louis, MO, USA).

### 2.4. Preparation of Stable Cell Lines

The Jurkat 2D10 T cell line model of HIV latency [63] has been extensively characterized and is widely utilized. The proviral gene in this model is derived from the NL4-3 isolate and contains the entire HIV 5′ LTR. It is modified to contain an EGFP reporter gene in place of Nef to allow for measurement of latent HIV reactivation via fluorescent detection of proviral gene expression. The inserted EGFP reporter has been further engineered to encode a protein (d2EGFP) with a short-lived half-life [63]. Jurkat 2D10 cells were infected with packaged viruses belonging to each of the MSCV FLAG-Tat constructs and 48 h later, treated with 3 µg/mL puromycin to select for the transduced cells. After three weeks of culture in puromycin-containing media, successful stable expression of FLAG-Tat was determined by Western blotting using both anti-FLAG and Tat antibodies.

### 2.5. Flow Cytometry

Following the stable ectopic expression of FLAG-Tat variants in Jurkat 2D10 cells, viral reactivation and cell viability were examined by flow cytometry measurements using the BD LSR Fortessa instrument and BD FACSDiva software v9.7.1 (BD Biosciences, Milpitas, CA, USA) with the appropriate voltage settings. Approximately 4 × 10^4^ cells were placed in 200 µL 1× PBS and then stained with 1 µL of 1 mg/mL propidium iodide (Cayman Chemical). Afterwards, cells were subjected to flow cytometry for the simultaneous analysis of LTR-driven reporter EGFP expression and cell viability. At least 10,000 events were captured for each cell sample. The resulting raw flow data in FCS file format were analyzed using Winlist 3D version 9.0.1 and the processed data with the appropriate gating were presented as either 2D scatterplots (EGFP vs. cell viability) or 1D histograms. Extent of EGFP expression was reported as a percentage of cells determined to be fluorescently positive relative to the appropriate gating control (uninfected Jurkat E6 cells).

### 2.6. Immunoprecipitation and Western Blotting

Harvested Jurkat or HEK 293 cells were washed once with 1× PBS then resuspended in cell lysis buffer A (150 mM NaCl, 10 mM KCl, 1.5 mM MgCl2, 0.5% NP-40, 1 mM DTT, 10 mM Hepes, pH 8.0) containing an EDTA-free protease and phosphatase inhibitor mixture (Roche Applied Science, Indianapolis, IN, USA). Whole-cell extracts were cleared by microcentrifugation at 2300× *g* for 5 min then incubated overnight at 4 °C with anti-FLAG M2 affinity resin (Millipore-Sigma) or anti-HA agarose beads (ThermoFisher Scientific, Waltham, MA, USA) on a microcentrifuge tube rotator (30 µL of packed beads per mL of whole-cell extracts (WCEs)). Immunoprecipitates were washed extensively with lysis buffer A and afterwards incubated overnight at 4 °C with elution buffer containing either FLAG or HA peptide at 200 µg/mL. Eluates, obtained by microcentrifugation at 2300× *g* for 2 min, as well as whole-cell extracts were boiled in 1× LDS sample loading buffer containing 50 mM DTT. SDS-PAGE was performed on 4–12% Bis Tris gels in MOPS or MES buffer at 150 V for 70 min, followed by transfer of resolved proteins to PVDF membranes. Thereafter, Western blotting was performed with primary antibodies against FLAG, Tat, Cyclin T1 (CycT1), CDK9, HEXIM1, MEPCE and LARP7. The sources of these antibodies as well as other reagents used in the study are shown in Supplementary Table S1. Chemiluminescence images of the Western blots were captured using the ImageQuant LAS 4000 (Amersham Biosciences, Wilmington, DE, USA). The resulting TIFF images were subjected to densitometry analysis using Quantity One software v4.6.8 (Bio-Rad, Hercules, CA, USA). A source data file of all original Western blots is provided as a separate file, complete with molecular weight ladders and clear annotations. In instances where membranes were cut horizontally or vertically to probe for multiple proteins of differing molecular weights, individual membrane strips are explicitly labeled and aligned with their corresponding molecular weight markers.

### 2.7. In Vitro Kinase Assay

Cleared whole-cell extracts prepared from Jurkat T cells belonging to untransduced controls or FLAG-CDK9 WT were subjected to anti-FLAG immunoprecipitation by overnight incubation at 4 °C with anti-FLAG M2 affinity resin (Millipore-Sigma). To assess CDK9 kinase activity, the anti-FLAG IP was divided equally into four microcentrifuge tubes and 50 µL of in vitro kinase reactions were set up as described previously [12], with or without a 15 min pre-incubation with increasing concentrations of flavopiridol (FVP; 25 to 100 nM). Following pre-incubation with FVP, 250 ng of His-tagged full-length human RNAP II CTD substrate (Abcam, Waltham, MA, USA) was added to each reaction set-up. The kinase assay was performed at 30 °C for 1 h. Reactions were terminated by boiling samples in 1× LDS sample loading buffer containing 50 mM DTT. Boiled samples were subjected to SDS-PAGE and the dried gel was analyzed by autoradiography.

### 2.8. Combined Immunofluorescence and RNA FISH Staining

Primary CD4^+^ T cells were harvested by centrifugation at 450× *g* for 3 min, washed with 1× PBS and resuspended in 30 mL 1× PBS. The cell suspension was placed onto poly-L-lysine-coated coverslips in a 24-well plate and allowed to adhere to the surface at 37 °C for 15 min. Cells were then fixed with 4% formaldehyde for 15 min, washed with 1× PBS and then permeabilized with a sequential treatment of 0.2% Triton-X-100 for 10 min and 1× BD Perm/Wash buffer (BD Biosciences) for 30 min at room temperature. For the blocking step, cells were incubated in blocking solution for 30 min made up of 1% BSA (10 µg/mL) and 50 µg/mL Donkey IgG in 1× Perm/Wash. Thereafter, cells were incubated with primary antibody solutions prepared in blocking buffer for 2 h at room temperature, washed three times with 1× Perm/Wash buffer then incubated with 1:200 dilutions of Alexa Fluor-conjugated anti-rabbit secondary antibodies that were also prepared in blocking buffer. After immunostaining, the coverslips were washed twice with 1× PBS and re-fixed in 4% formaldehyde solution prior to initiation of the RNA FISH procedure. Coverslips were washed once with 1× PBS before incubation with Wash Buffer A (2× saline-sodium citrate (SSC) and 10% formamide in RNase-free ultrapure water) for 5 min at room temperature. For RNA FISH, Stellaris custom-synthesized fluorophore-conjugated oligonucleotide probe sets (Biosearch Technologies, Alexandria, MN, USA; Supplementary Table S2) were resuspended in hybridization buffer (10% dextran sulfate, 2× SSC and 10% formamide in RNase-free ultrapure water) to a final concentration of 375 nM. Coverslips were inverted onto 40 μL droplets of the hybridization mixture in a humidified chamber which was then tightly sealed and incubated in the dark at 37 °C for 16 h. Afterwards, coverslips were transferred onto a fresh 24-well plate cells side up, incubated in the dark with Wash buffer A for 30 min and then counterstained for 15 min with 1 μg/mL DAPI prepared in Wash Buffer A. Coverslips were washed twice with Wash Buffer B (2× SSC in RNAase-free ultrapure H_2_O) prior to mounting onto glass slides using Prolong Antifade medium. Images were captured at high magnification (100×) by deconvolution microscopy using a DeltaVision epifluorescent microscope (Applied Precision). Raw *z* stack images were deconvolved and processed using the softWoRx analysis program (Applied Precision). Deconvolved images (D3D files) were scaled identically across treatment conditions. For colocalization measurements, the softWoRx colocalization module was used to create correlation scatterplots and measure the Pearson’s coefficient. A region of interest for the colocalization analysis was defined as a single cell. Processed images were exported as JPEG files and micrographs were composed using Adobe Photoshop v26.8.1. The resulting TIFF files were confirmed to have preserved the full fluorescence signal data in each channel (FITC, Cy5, and DAPI) for each treatment condition. Fluorescence densitometry was subsequently performed on these uncompressed files using Quantity One software v4.6.8 (Bio-Rad).

### 2.9. Statistical Analysis

Box-and-whisker plots for flow cytometry d2EGFP reporter data were prepared in Origin v10.0.5.157 (OriginLab Corp, Northampton, MA 01060). Microsoft Excel was used to generate bar graphs for the Western blotting and cell viability measurements. Error bars were generated from at least three independent experiments with findings presented as mean +/− standard error. Comparisons between two experimental groups were conducted using a two-tailed Student’s *t*-test. Comparison between and within multiple groups was determined by a one-way ANOVA with a post hoc two-tailed Student’s *t*-test.

## 3. Results

### 3.1. Tat:7SK Is an HEXIM1-Deficient Reversible Complex That Releases Tat:P-TEFb

**Figure 1:**
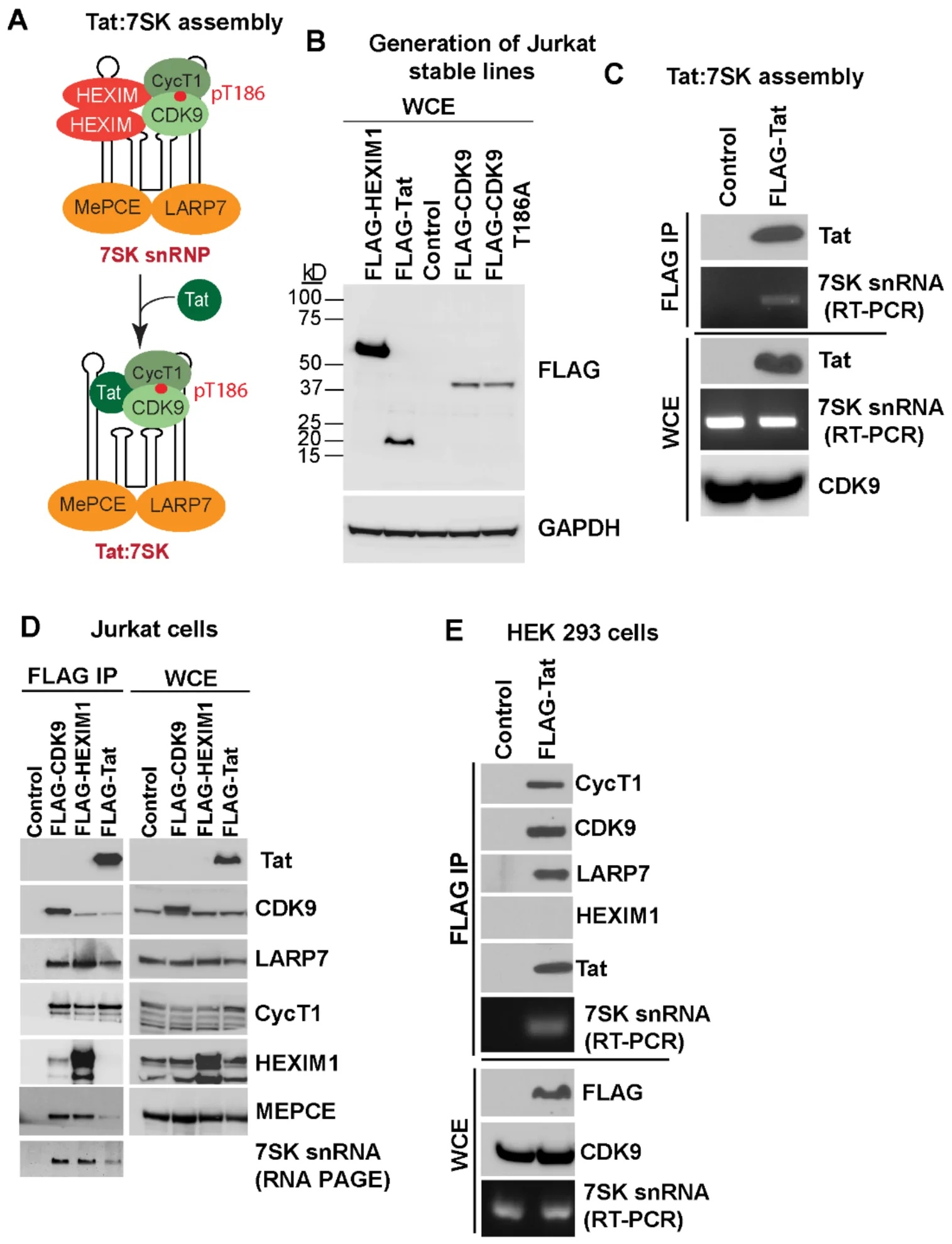
Tat incorporates into 7SK snRNP to form an HEXIM1-deficient ribonucleoprotein complex. (**A**) Scheme of Tat:7SK assembly and Tat displacement of HEXIM1. (**B**) Stable ectopic expression in Jurkat 2D10 cells of FLAG-tagged proteins utilized in the current study. Whole-cell extracts (WCEs) were analyzed by Western blotting using an FLAG or a GAPDH antibody. (**C**–**E**) Immunoprecipitation (IP) of FLAG-Tat from Jurkat T cells or HEK 293 cells shows formation of Tat:7SK with the complete displacement of HEXIM1. In Panel (**D**), immunoprecipitations of FLAG-CDK9 and FLAG-HEXIM1 served as positive controls for 7SK snRNP co-precipitation. In (**C**–**E**), whole-cell extracts (WCEs) were subjected to affinity IP with anti-FLAG M2 agarose beads prior to elution with FLAG peptide. Both eluates and WCE were analyzed by Western blotting for the protein components shown. RNA was also extracted from IP and WCE fractions and subjected to either RT-PCR or denaturing RNA PAGE to detect the presence of 7SK snRNA. The slight shift in the migration patterns of FLAG-CDK9 and FLAG-HEXIM1 observed in the Panel (**D**) blots is due to immunodetection of both the endogenous and FLAG-tagged forms of the proteins.

**Figure 2:**
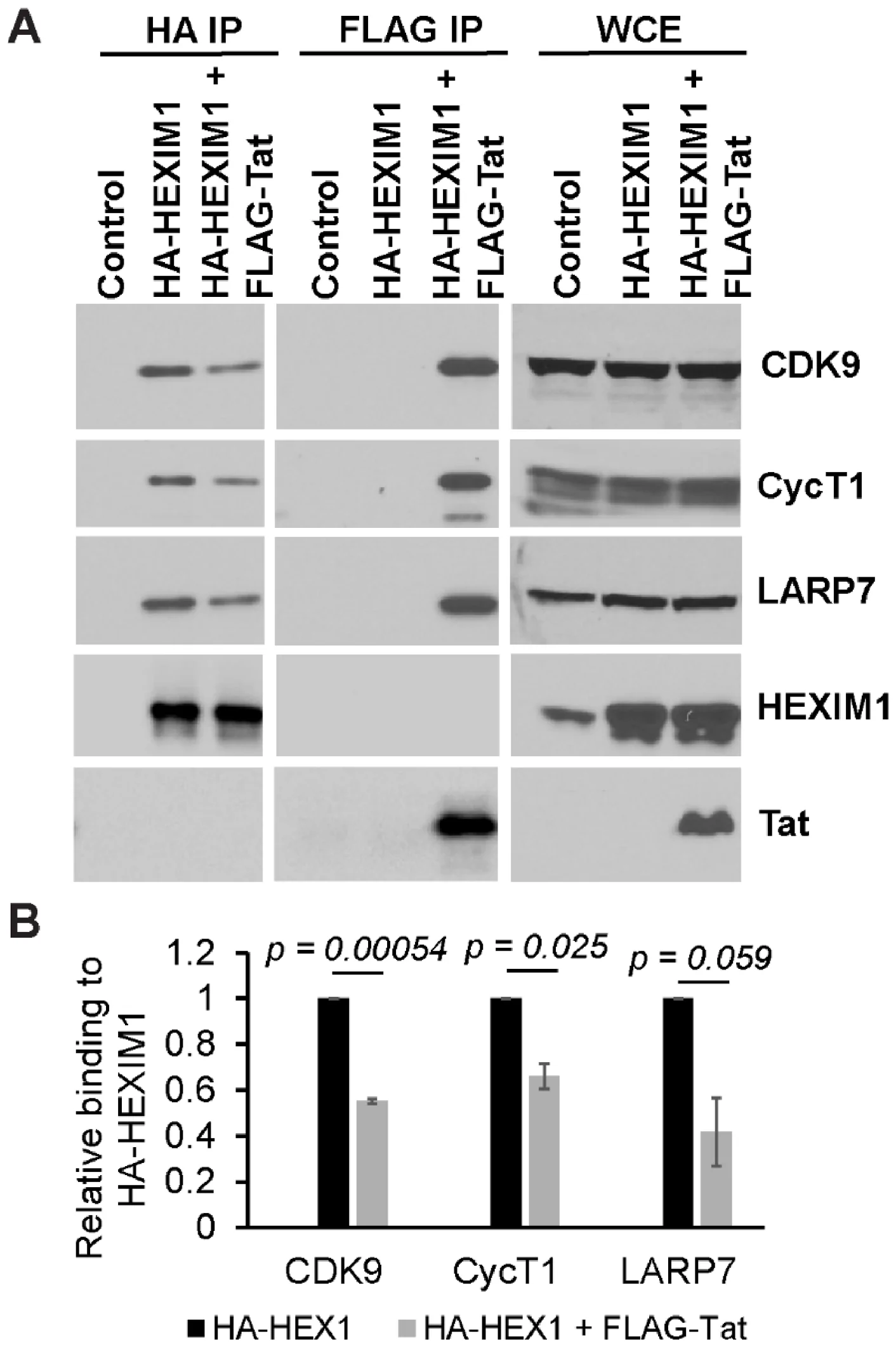
Reciprocal immunoprecipitation confirms that Tat and HEXIM1 are mutually exclusive within the 7SK snRNP. (**A**) HEK 293 cells stably expressing HA-tagged HEXIM1 were transiently transfected with FLAG-Tat. Whole-cell extracts (WCEs) were subjected to IP with anti-FLAG or anti-HA antibody followed by elution with FLAG or HA peptide, respectively. Both eluates and WCE were analyzed by Western blotting for the protein components shown. (**B**) Densitometry quantification of the extent of CDK9, Cyclin T1 (CycT1) and LARP7 association with HEXIM1 with or without the transient transfection of FLAG-Tat. Graphed data are from three independent IP experiments. Statistical significance was determined using a two-tailed Student’s *t*-test.

Tat is well established to bind 7SK snRNP while simultaneously promoting the release of P-TEFb from this sequestrator complex [59,64,65]. However, it remains unclear whether Tat:7SK is a biologically relevant intermediary complex that mediates the reversal of P-TEFb sequestration. The initial report characterizing the assembly of Tat:7SK defined it as a ‘Tatcom2’ complex lacking the P-TEFb inhibitory partner HEXIM1 (Figure 1A) [59]. The study further described Tat:7SK as being stably resistant to dissociation by stimuli, stress signals and agents known to disrupt 7SK snRNP. Other reports have characterized Tat:7SK as a complex that maintains HEXIM1, whose displacement and subsequent liberation of P-TEFb is facilitated by Tat’s recruitment of an E3 ubiquitin ligase UBE2O or the protein phosphatase PPM1G [66,67]. To ascertain the composition of Tat:7SK, affinity tag-based immunoprecipitation (IP) studies were carried out using whole-cell extracts either belonging to Jurkat T cells stably expressing an N-terminally FLAG-tagged form of clade B NL4-3 Tat (Figure 1B–D), or HEK 293 cells transiently transfected with the same FLAG-Tat construct (Figure 1E). Transient transfection of HEK 293 cells with FLAG-Tat was intended to mimic a state of Tat expression that occurs during acute HIV infection or upon the reactivation of latently infected cells. Anti-FLAG IP followed by elution of bound complexes with FLAG peptide confirmed the assembly of Tat:7SK with complete displacement of HEXIM1 in both the Jurkat stable cell line as well as the transiently transfected HEK 293 cells (Figure 1C,D). Immunoprecipitations of FLAG-CDK9 and FLAG-HEXIM1 served as positive controls for 7SK snRNP co-precipitation (Figure 1D). Since all known protein components of 7SK snRNP (CDK9, CycT1, HEXIM1, LARP7 and MEPCE) remain stably and constitutively expressed in actively dividing cells [13,41,68,69], any of them can reliably serve as reliable input controls for the IP experiments. In HEK 293 cells stably expressing HA-HEXIM1, transient expression of FLAG-Tat promoted Tat:7SK complex assembly lacking HEXIM1 while partially disrupting the interaction between HA-HEXIM1, P-TEFb, and LARP7 (Figure 2). Conversely, in cells expressing FLAG-Tat, immunoprecipitation of HA-HEXIM1 showed that its 7SK snRNP fraction does not contain Tat (Figure 2). These results confirm that Tat and HEXIM1 exclude each other from 7SK snRNP.

Because Tat physically removes P-TEFb from 7SK snRNP but also forms an HEXIM1-deficient Tat:7SK complex, we sought to determine if Tat:7SK is dissociable, potentially serving as an exchange intermediate. Since both LARP7 and MEPCE constitute the integral, ribonucleoprotein core required to stabilize the 7SK snRNA scaffold, 7SK snRNA cannot form an RNP assembly in the absence of these two proteins [68,70,71,72,73]. Furthermore, because neither Tat nor P-TEFb form direct protein–protein contacts with LARP7 or MEPCE [59,74,75,76], Tat’s association with LARP7 and MEPCE is strictly dependent on, and mediated through, the intact 7SK snRNA scaffold itself. Thus, tracking the co-precipitation of Tat with LARP7 and MEPCE serves as a robust biochemical surrogate for the overall Tat:7SK complex integrity, even when facultative components like HEXIM1 are displaced.

**Figure 3:**
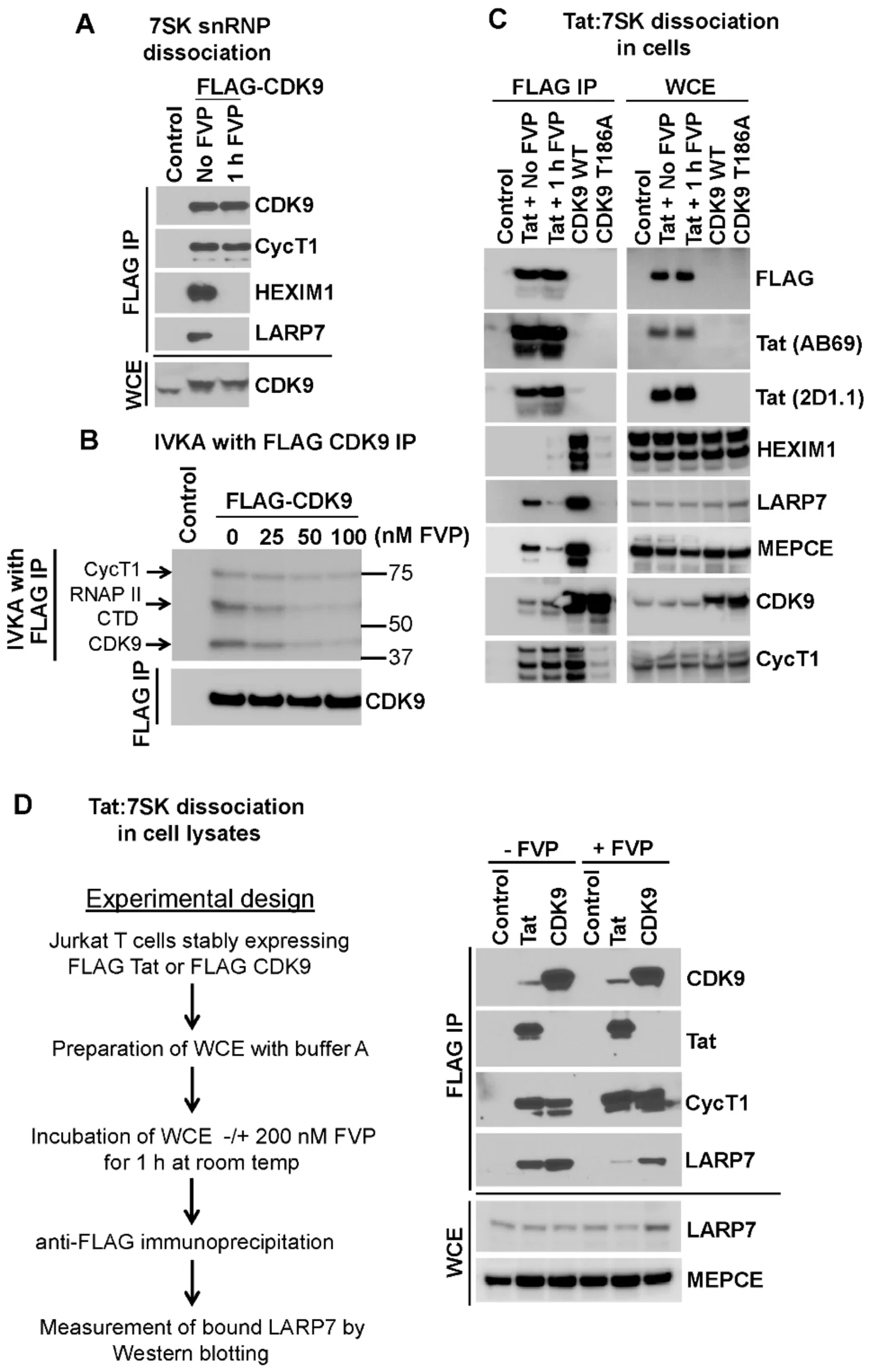
Tat reversibly interacts with 7SK snRNP. (**A**) Rapid dissociation of 7SK snRNP to release P-TEFb upon treatment of Jurkat 2D10 T cells stably expressing FLAG-CDK9 with flavopiridol (FVP). The slight shift in the migration pattern of FLAG-CDK9 observed in the WCE blot is due to immunodetection of both the endogenous and FLAG-tagged form of the protein. (**B**) Radioactive in vitro kinase assay performed using an anti-FLAG-CDK9 IP with the addition of a His-tagged C-terminal domain (CTD) RNAP II substrate to assess CDK9 kinase activity in the presence or absence of in vitro treatment with increasing concentrations of FVP. The arrows point to bands representing autophosphorylated CDK9, phospho-CycT1 and phospho-RNAP II CTD. (**C**) Rapid dissociation of Tat:7SK in FLAG-Tat-expressing 2D10 cells by FVP. This experiment confirms that Tat:7SK contains P-TEFb. In (**A**,**C**), cells were treated or not with 100 nM FVP for 1 h. Whole-cell extracts (WCEs) were subjected to IP with anti-FLAG M2 agarose beads followed by elution with FLAG peptide. Both eluates and WCE were analyzed by Western blotting for the protein components shown. An anti-FLAG antibody, 2D1.1 mouse monoclonal antibody targeting resides 2–15 of Clade B Tat, and a rabbit polyclonal antibody (AB69) were used to detect Tat. (**D**) FVP treatment of Jurkat 2D10 cell extracts causes the dissociation of 7SK snRNP and Tat:7SK. As shown in the scheme, WCE belonging to cells stably expressing FLAG-Tat or FLAG-CDK9 were treated or not with 200 nM FVP for 1 h then subjected to anti-FLAG IP. FLAG eluates were analyzed by immunoblotting for the proteins shown. Association with 7SK snRNP was determined by assessing the levels of co-precipitated LARP7.

CDK9 kinase inhibitors, such as flavopiridol (FVP), rapidly and almost completely dissociate intracellular 7SK snRNP complexes (Figure 3A) [42,44,54], though the precise mechanisms remain unclear. In our cell-based assessments, FVP was utilized at 100 nM, a concentration that is known to effectively inhibit CDK9 kinase activity (Figure 3B) [54]. Our previous work has shown that treatment of Jurkat T cells with increasing concentrations of FVP (25 nM to 100 nM) leads to a dose-dependent dissociation of P-TEFb from HEXIM1 [13]. In the same study, we also found that treatment of Jurkat T cells with 100 nM FVP does not affect the expression of either phospho-Thr186 CDK9 or phospho-Ser175 CDK9 up to 6 h post-treatment with the inhibitor. Therefore, the rapid dissociation of 7SK snRNP due to FVP cannot be attributed to dephosphorylation of CDK9 at Thr186 or Ser175.

Treatment of Jurkat cells stably expressing FLAG-Tat with 100 nM FVP for 1 h resulted in a near-complete disruption of Tat’s association with the core 7SK snRNP subunits MEPCE and LARP7 (Figure 3C). By contrast, Tat’s interaction with P-TEFb subunits was unaffected by FVP treatment. Since the T186A CDK9 mutation disrupts P-TEFb assembly and cannot be incorporated into 7SK snRNP [13], it served as a crucial control to validate the specificity of co-immunoprecipitated interactions between FLAG-Tat or FLAG-CDK9 WT. Kinase and phosphatase inhibitors known to trigger the release of P-TEFb from 7SK snRNP are thought to do so by inducing stress pathways [42,44,51]. To investigate whether FVP disrupts 7SK snRNP and Tat:7SK complexes directly rather than through the activation of stress signaling, whole-cell extracts belonging to Jurkat cells stably expressing FLAG-Tat or FLAG-CDK9 were treated with FVP for 1 h prior to anti-FLAG IP. Immunoblotting of the FLAG eluates revealed a substantial loss of P-TEFb or Tat association with LARP7 upon treatment with FVP (Figure 3D). These results suggest that FVP binding to CDK9 triggers the dissociation of both 7SK snRNP and Tat:7SK complexes. Our findings support the observation by Sobhian et al. that Tat:7SK contains P-TEFb and is missing HEXIM1 [59]. However, contrary to their model defining Tat:7SK as a dissociation-resistant complex, our data reveal it to be a dynamic, reversible complex which is responsive to a CDK9 kinase inhibitor. Thus, the *en bloc* release of Tat:P-TEFb shows that Tat:7SK can function as an intermediate for P-TEFb mobilization.

### 3.2. Clade Variations in Tat:7SK Assembly and P-TEFb Mobilization

**Figure 4:**
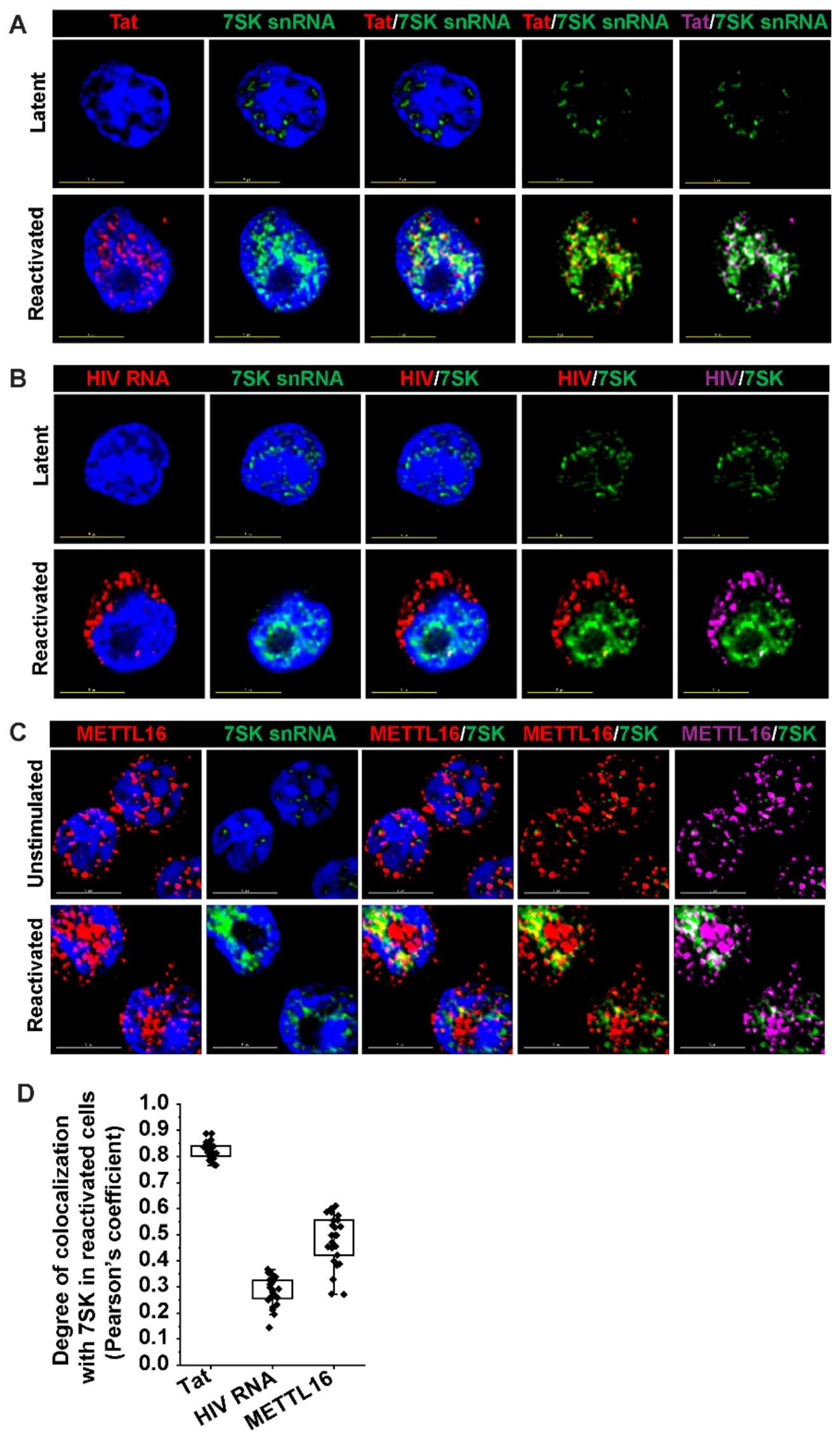
Strong nuclear colocalization of Tat with 7SK snRNP in infected primary T cells. (**A**) Combined immunofluorescence staining for Tat and 7SK snRNA FISH in latent and reactivated QUECEL primary Th17 cells. (**B**) Detection of total HIV RNA and 7SK snRNA by FISH in latent and reactivated primary Th17 cells. Total HIV RNA transcripts were detected using a viral probe set which hybridizes to the RNA region encoded by the HIV LTR. (**C**) Combined immunofluorescence staining for METTL16 and 7SK snRNA FISH in unstimulated and reactivated memory CD4^+^ T cells. Scale bars in A–C indicate a distance of 5 µm. Panels with magenta/green pseudocoloring are presented to ensure optimal distinction for readers with color vision deficiencies. (**D**) Graph comparing the degree of colocalization with 7SK in reactivated cells.

Demonstrating the existence of Tat:7SK in HIV-infected primary CD4^+^ T cells is crucial but challenging due to the difficulty of obtaining sufficient cells for immunoprecipitation assays. To circumvent this, we have opted to employ combined immunofluorescence staining and RNA FISH for high-resolution microscopy which consistently revealed a strong nuclear colocalization of Tat with 7SK snRNA upon the reactivation of latent HIV in a primary Th17 cell model of infection (Figure 4). The IF-RNA FISH imaging in Figure 4B,C (HIV RNA/7SK snRNA and METTL16/7SK snRNA) were performed as essential spatial controls to establish the specificity of the subnuclear colocalization observed between Tat and 7SK snRNA. Unlike Tat and 7SK snRNA, which displayed tight, coincident nuclear colocalization, reactivated viral transcripts (HIV RNA) localized almost exclusively to the cytoplasm upon 24 h of TCR activation (Figure 4B). This distinct spatial separation demonstrates that the subnuclear overlap seen in Figure 4A is specific to Tat/7SK and not an artifact of generalized cellular activation or fluorescence bleed-through. METTL16 is a known m6A methyltransferase that has been shown to interact with 7SK snRNA [77]. While METTL16 exhibited a partial subnuclear overlap with 7SK snRNA, its signal was broadly distributed across the nucleus and lacked the dense, doughnut-shaped coincidence observed between Tat and 7SK snRNA. Moreover in the activated primary T cells, METTL16 appears to concentrate in the nucleolar region, an observation consistent with its role in facilitating ribosomal biogenesis [78,79]. This comparison underscores the high spatial specificity of the Tat/7SK colocalization during latent viral reactivation. Definitively demonstrating Tat:7SK assembly in infected primary T cells via fluorescence-based imaging necessitates proximity labeling, which we aim to implement in the near future.

**Figure 5:**
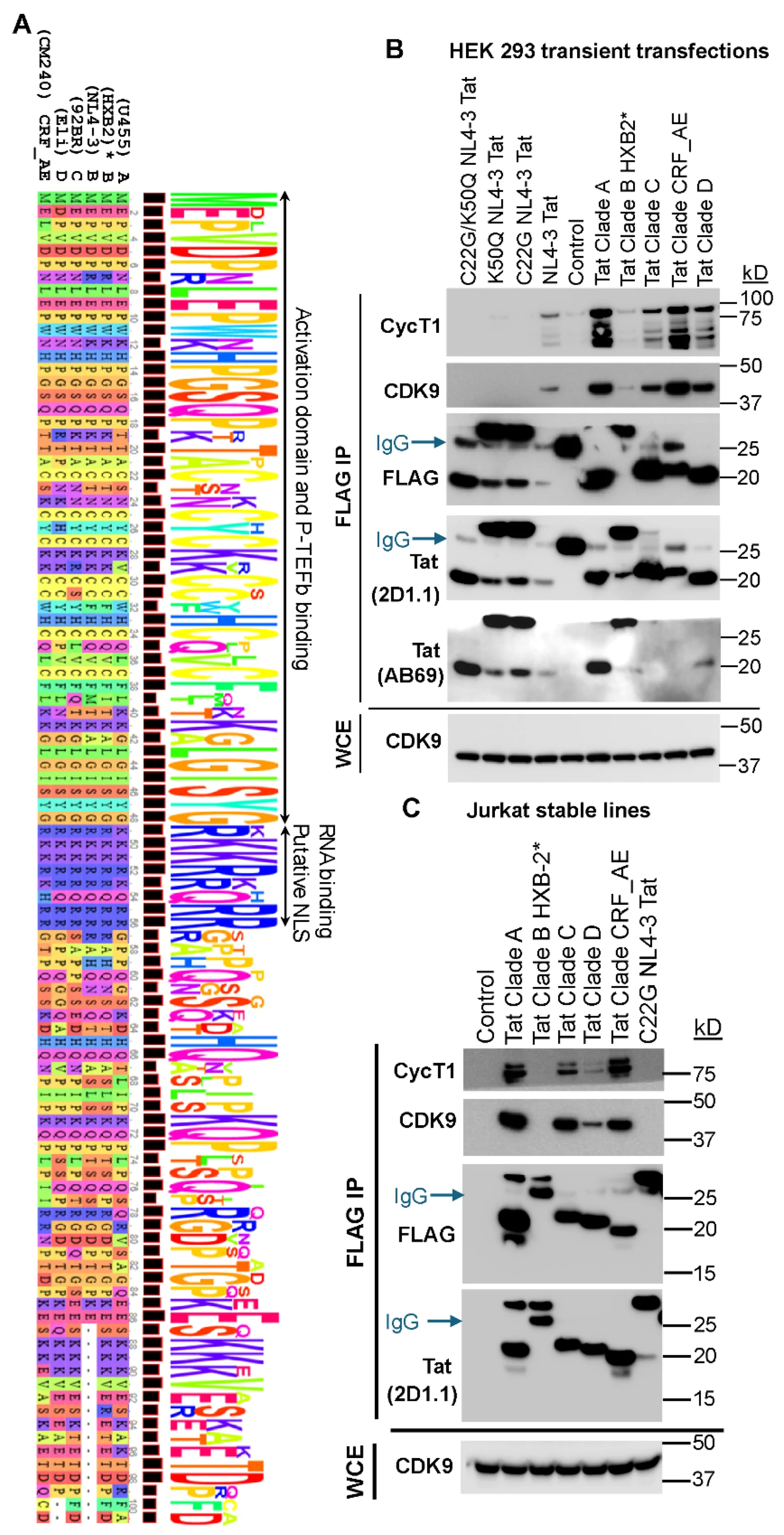
Ectopic expression of clade-representative Tat variants. (**A**) Sequence alignment and origin of clade-representative Tat sequences used in the current study. N-terminally FLAG-tagged constructs of these Tat variants were generated and either transiently transfected into HEK 293 cells or stably expressed in Jurkat cells. (**B**,**C**) Detection of the ectopic expression of the Tat variants in HEK 293 or Jurkat cells using three different antibodies: FLAG antibody; the 2D1.1 monoclonal antibody whose binding epitope corresponds to residues 2–15 of Tat; and a polyclonal antibody (AB69) whose binding epitope corresponds to residues 2–22 of Tat. The blue arrows in (**B**,**C**) point to bands emanating from the IgG light chain component of anti-FLAG used for IP, that carried over into the FLAG eluate fraction. (HXB2)* denotes the full-length HXB2 Tat variant.

After ascertaining that Tat:7SK assembly is reversible and likely exists in infected primary T cells, we next explored whether genetic variations in Tat across HIV clades influence its capacity to interact with 7SK snRNP, mobilize P-TEFb, and transactivate latent HIV. Figure 5A shows an alignment and origin of clade-representative Tat sequences that were used in our study, which show sequence differences in the N-terminal P-TEFb-binding region. We used a full-length version of the reference HXB2 Tat sequence, in which we mutated the premature stop codon back to a conserved serine. N-terminally FLAG-tagged constructs of these Tat variants were generated and either transiently transfected into HEK 293 cells or stably expressed in Jurkat cells. To detect the ectopic expression of these Tat variants, we utilized three distinct antibodies: (a) an anti-FLAG antibody; (b) a custom-synthesized 2D1.1 mouse monoclonal antibody targeting residues 2–15 of clade B Tat [80]; and (c) a custom-synthesized polyclonal antibody (AB69) whose binding epitope corresponds to residues 2–22 of clade B Tat.

Despite minor clade variations within residues 2–15 across clades, the 2D1.1 monoclonal antibody detected all examined Tat variants as effectively as the FLAG antibody (Figure 5B,C). This was in contrast to the AB69 antibody which only detected Clade A Tat as well as the NL4-3 variant (Figure 5B). Unexpectedly, the full-length HXB2 Tat migrated at a higher molecular weight of ~28 kD, likely representing an SDS-resistant dimer, under denaturing and reducing SDS-PAGE conditions (Figure 5B,C). This higher molecular weight form was also observed with the NL4-3 Tat C22G and K50Q single-point mutants, detectable by either 2D1.1 or AB69 antibody, but was absent in wild-type NL4-3 Tat. Interestingly, double mutation of Cys-22 and Lys-50 (C22G/K50Q) restored the SDS-PAGE migration to the expected monomeric form when probed with either the 2D1.1 and AB69 antibody (Figure 5B). There are several reports of HIV-1 Tat primarily migrating as a dimer during SDS-PAGE because it forms highly stable, covalent-like complexes that resist complete denaturation by SDS and boiling under reducing conditions [81,82,83]. This behavior is apparently driven by its unique cysteine-rich domain which tightly binds divalent cations such as Zn2+ and creates intermolecular disulfide bonds between monomers. Since Tat oligomerization likely hinders interactions with host factors such as P-TEFb—a finding supported by immunoprecipitation data for full-length HXB2 Tat (Figure 5B,C)—we chose to use the wildtype NL4-3 Tat as the clade B representative. This allowed an effective comparison of intracellular Tat interactions with 7SK snRNP and P-TEFb, as well as the ability to transactivate latent HIV in Jurkat stable lines prepared using the 2D10 clonal model.

**Figure 6:**
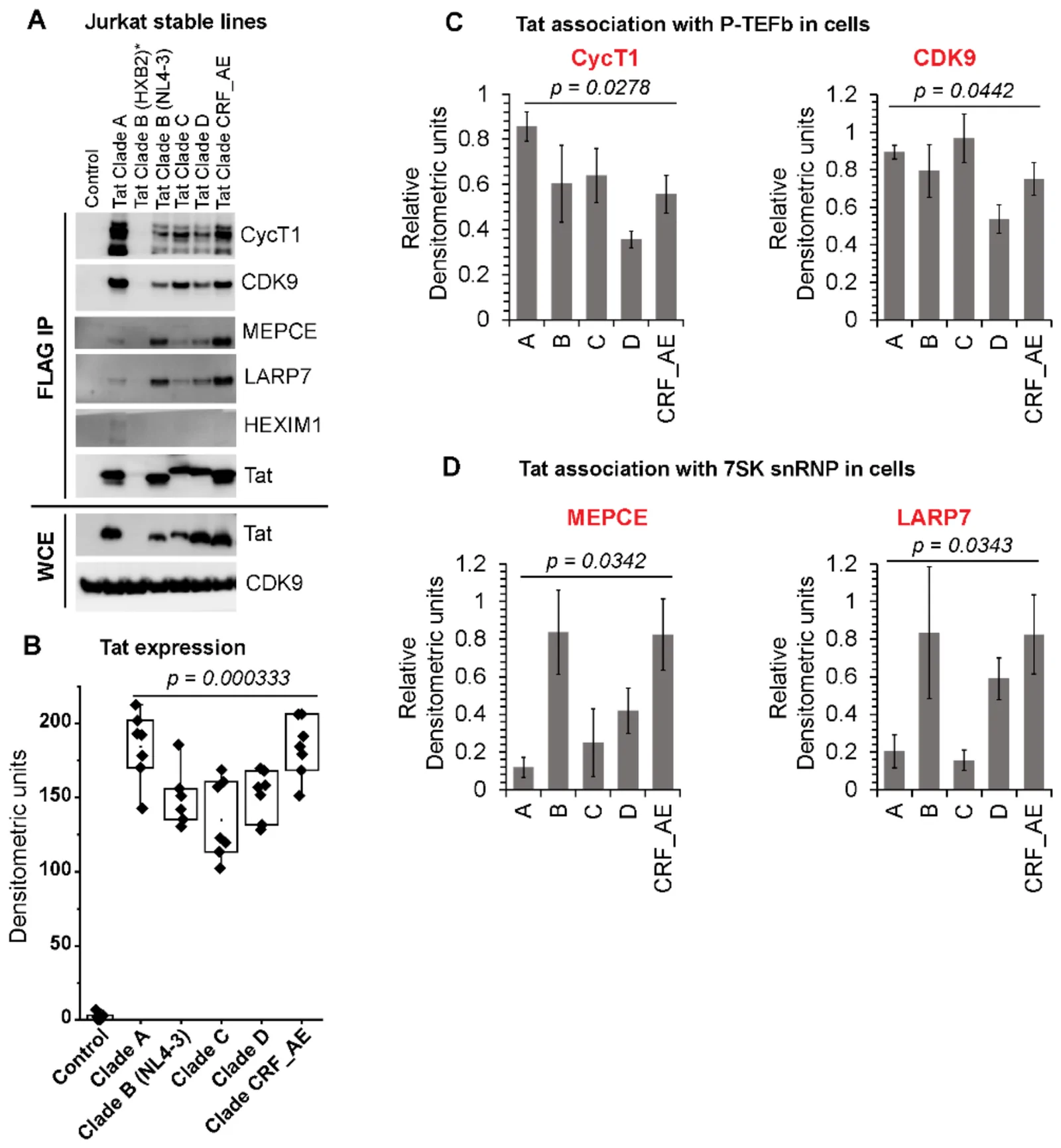
Clade A Tat efficiently mobilizes P-TEFb from 7SK snRNP. (**A**) Extent of Tat association with P-TEFb and 7SK snRNP protein components. (**B**) Comparison of the ectopic expression of Tat variants in Jurkat stable lines. (**C**,**D**) Densitometric quantification of co-precipitated proteins after normalization to corresponding immunoprecipitated Tat. Quantification of Tat association with P-TEFb, MEPCE or LARP7 across the representative clade variants. Plotted data are from at least three independent IP experiments. Statistical significance was determined by a one-way ANOVA with a post hoc Student’s *t*-test. *p* values from the post hoc *t*-test are presented in Supplementary Table S3. (HXB2)* denotes the full-length HXB2 Tat variant.

Stable transduction of FLAG-Tat clade variants in Jurkat 2D10 cells resulted in variable Tat protein expression. Clades A and CRF_AE yielded similar expression levels, which were significantly higher than the comparable levels of clades B, C and D (Figure 6A,B; Supplementary Table S3). Normalization of co-precipitated proteins to the corresponding expression levels of Tat (Figure 6B) revealed statistically significant differences in the extent of P-TEFb binding and association with the 7SK snRNP core components MEPCE and LARP7, as determined by a one-way ANOVA with a post hoc Student’s *t*-test (Figure 6C,D; Supplementary Table S3). For comparative reference, levels of co-precipitated proteins without normalization to Tat expression are shown in Supplementary Figure S1; corresponding statistical data are presented in Supplementary Table S4. Most notably, A and C Tat showed a robust interaction with P-TEFb, significantly higher than D Tat, but exhibited the least association with MEPCE and LARP7 (Figure 6C,D). If the major source of cellular P-TEFb in Jurkat T cells is 7SK snRNP, these IP data suggest that A and C Tat are more efficient than the other variants examined at reversing the sequestration of the enzyme.

### 3.3. Clade Variations in Tat Transactivation of Latent HIV

**Figure 7:**
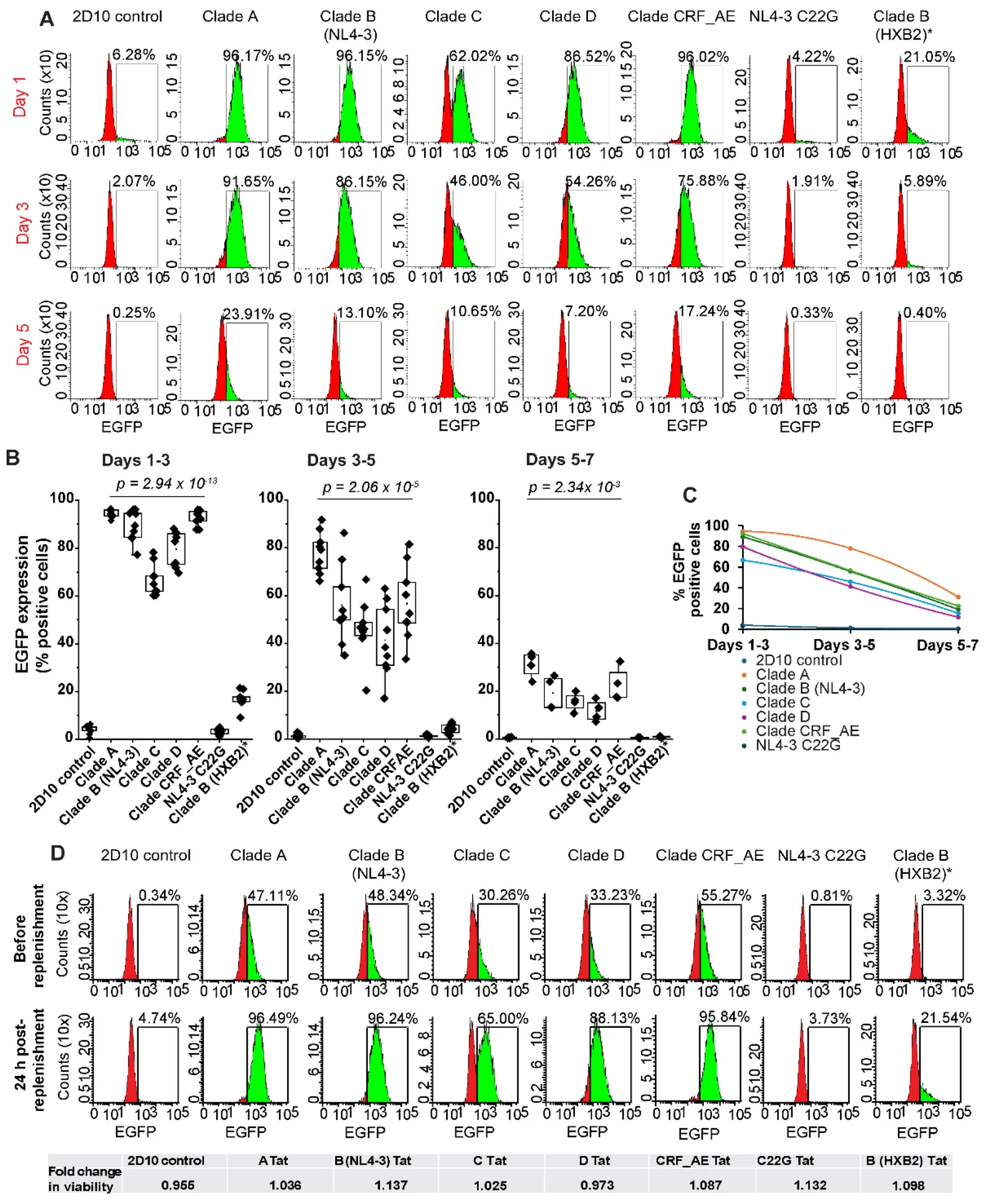
Cellular restriction of Tat varies by clade, creating differences in ability to transactivate latent HIV. (**A**) Representative flow plots showing spontaneous proviral reactivation in Jurkat 2D10 cells ectopically expressing FLAG-Tat variants and the temporal loss of Tat transactivation capacity. (**B**,**C**) Composite data from multiple independent replicates showing initial peak viral reactivation following a 1:3 media replenishment and variations in the diminution of Tat transactivation capacity. Graph in (**C**) is a fitted polynomial plot of the averaged values. Statistical significance was determined by a one-way ANOVA with a post hoc Student’s *t*-test. *p* values from the post hoc *t*-test are presented in Supplementary Table S3. (**D**) Peak viral reactivation upon culture media replenishment at a 1:3 ratio without a change in cell viability. In A and D, the EGFP positive cell population is shown in green. (HXB2)* denotes the full-length HXB2 Tat variant.

The varying efficiency with which Tat binds P-TEFb and extracts it from 7SK snRNP may confer differences in Tat’s ability to transactivate latent HIV. We examined the extent of viral reactivation using the Jurkat 2D10 clonal model. This model carries a latent NL4-3 provirus in which the expression of a short-lived d2EGFP reporter is driven by the HIV LTR [63]. Stable transduction of FLAG-Tat clade variants in Jurkat 2D10 cells triggered spontaneous latent HIV reactivation (Figure 7A). The experimental scheme for data shown in Figure 7 is presented in Supplementary Figure S2. Viral reactivation peaked 24 h after a 1:3 media replenishment (addition of 3 parts of media to 1 part of cell suspension) but progressively declined, reaching its lowest point 5 to 7 days post-replenishment (Figure 7A–C). Reactivation could be fully restored by repeating the 1:3 media exchange (Figure 7D). Notably, these cyclic fluctuations in Tat activity were independent of changes in cell viability (Figure 7D and Supplementary Figure S3).

At peak Tat activity (Days 1 to 3 post-replenishment), Tat from clades A, B, D and CRF_AE elicited comparable degrees of viral reactivation, whereas clade C Tat induced the lowest levels (Figure 7A,B). Because this pattern in peak LTR-driven d2EGFP expression mirrored ectopic Tat protein levels (Figure 6B), it suggested that early viral reactivation may have been driven by Tat expression differences between the clades. However, although clade D Tat initially induced significantly higher peak viral reactivation than clade C Tat, it experienced a steeper temporal decline, ultimately dropping its minimal transactivation capacity to below that of clade C Tat (Figure 7B,C; Supplementary Table S5). Consequently, the most significant disparity in the temporal loss of Tat activity was observed between clades A and D (Figure 7C). Overall, these results demonstrate that cellular restriction of Tat varies by clade—particularly between A and D—causing differences in its ability to transactivate latent HIV.

## 4. Discussion

Releasing P-TEFb from the 7SK snRNP complex is crucial for activating latent retroviral transcription, including HIV-1 and HTLV-1. To stimulate efficient transcription elongation, the HIV-1 Tat protein recruits P-TEFb from 7SK snRNP to TAR RNA anchored at the promoter-proximal region of the provirus. Tat also interacts with various host chromatin-modifying enzymes to promote transcription initiation. Consequently, recent efforts using ectopic Tat as a selective latency-reversing agent (LRA) to minimize off-target cellular effects have bolstered the feasibility of the “Shock and Kill” strategy deliberately intended to reactivate latent HIV to clear persisting infected memory T cells [15,16,17]. This LRA approach would benefit from a detailed mechanistic understanding of Tat-mediated P-TEFb release from 7SK snRNP and the identification of Tat variants with superior transactivation activity. Notably, unlike HTLV-1 Tax and the host factor BRD4, which physically extract P-TEFb from 7SK snRNP without binding the RNA scaffold, Tat assembles with this RNP complex while simultaneously promoting P-TEFb release [55,59,64,65,84]. Our current study investigated whether Tat:7SK forms a reversible complex that acts as an intermediate for recruiting P-TEFb to proviral HIV. Furthermore, because geographically diverse HIV clades exhibit sequence variation in the protein–protein interaction domains of Tat, this study also evaluated whether clade-specific differences influence Tat’s ability to bind P-TEFb, assemble with 7SK snRNP, and transactivate latent HIV in infected cells.

### 4.1. Tat Forms a Reversible Complex with 7SK snRNP

Using combined immunofluorescence and RNA FISH, we confirmed that Tat strongly colocalizes with nuclear 7SK snRNA following the reactivation of latently infected QUECEL primary Th17 cells, consistent with prior reports [85]. These results indicate that the Tat:7SK complex likely exists in infected primary T cells, a premise that will require definitive demonstration by proximity labeling. Affinity isolation of Tat:7SK from transiently transfected HEK 293 cells and a Jurkat stable cell line confirmed the initial discovery by Sobhian et al. that this complex contains P-TEFb and lacks the inhibitory protein HEXIM1 [59]. These findings are also consistent with prior reports demonstrating that Tat displaces HEXIM1 from 7SK snRNP by competitively interacting with the 5′ hairpin (Stem Loop; SL1) of 7SK snRNA. In particular, NMR structural studies have revealed that the recognition of SL1 by the RNA binding motif of Tat is enabled by Arg-52, which remodels a pseudo arginine sandwich motif responsible for HEXIM1 binding into a classical configuration that causes the inhibitory protein to be ejected [56,58]. This structural analysis further demonstrated that although HEXIM1 binds SL1 with a high degree of specificity, albeit at a three- to four-fold lower binding affinity compared to Tat, it induces a local destabilization of the upper half of SL1 that is further exploited by Tat to bind 7SK snRNA more efficiently.

The Tat:7SK complex was previously described as a stable RNP assembly resistant to stimuli, stress signals, and agents that typically disrupt 7SK snRNP [59]. This permanence implied that Tat incorporation forms a “dead-end” complex, effectively sequestering P-TEFb from transcriptional control. Contrasting with this prevailing model, we demonstrate that Tat:7SK is sensitive to dissociation. Brief cellular treatment with flavopiridol (FVP), a selective P-TEFb kinase inhibitor that disrupts 7SK snRNP assembly, dissociated both Tat:7SK and 7SK snRNP in cells and in whole-cell extracts. Because small molecules that trigger P-TEFb release are generally thought to act indirectly via stress pathways [42,44,51], these results also reveal a direct disruptive mechanism for FVP. Consequently, our findings confirm the presence of P-TEFb within Tat:7SK, demonstrate that enzyme liberation is coupled to its interaction with Tat, and clarify that CDK9 inhibitors disrupt 7SK snRNP directly. Mechanistically, high-resolution X-ray structures show that small-molecule binding to the CDK9 ATP-active site induces substantial conformational changes. Specifically, conserved regions like the glycine-rich loop rearrange to deeply bury the inhibitor [86,87]. These ligand-induced changes alter the structural plasticity and domain orientation at the CDK9–CycT1 inter-subunit interface, a perturbation that likely propagates to disrupt the interaction surfaces required for 7SK snRNP association.

Although HIV clades vary in their array of proviral transcription factor binding sites and Tat sequences [88], transcriptional control is rarely compared across subtypes. Fundamental mechanistic models of latency regulation and Tat-mediated transactivation have relied almost exclusively on subtype B strains, most notably NL4-3, even though non-B subtypes account for over 85% of current global infections and an increasing proportion in the global North [89,90,91,92]. Genetic variations in Tat are critical because they alter protein expression levels and transactivation potency, shifting the stochastic switch that governs viral fate [93,94,95]. For instance, attenuated Tat mutants reduce viral transcription below a critical threshold, driving infected cells into stable latency rather than productive replication [95]. Consequently, Tat sequence variants and altered transactivation potency are predicted to influence the responsiveness to latency-reversing interventions and “Block and Lock” strategies [96,97,98].

### 4.2. Clade Variations in Tat:7SK Assembly, P-TEFb Mobilization and HIV Transactivation

Since Tat interacts with multiple host factors (7SK snRNP and chromatin-modifying enzymes) as well as viral TAR RNA, subtle genetic changes in Tat are likely to significantly alter viral transactivation initiation and elongation feedback loops. We therefore tested the hypothesis that Tat clade variants differ in their ability to assemble with 7SK snRNP, mobilize P-TEFb and transactivate latent HIV. Stable transduction of N-terminally FLAG-tagged Tat variants in Jurkat 2D10 cells produced variable protein expression. Constructs from representative variants of clades A (U455) and CRF_AE (CM240) yielded similar expression levels which were significantly higher than the comparable levels of clades B (NL4-3), C (92BR) and D (Eli). Unsurprisingly, peak spontaneous HIV reactivation in Jurkat 2D10 cells—in which the expression of a short-lived d2EGFP reporter is driven by the HIV LTR—mirrored the differential ectopic expression of the variants, with clade C Tat displaying the lowest transactivation potential. These results are consistent with prior observations that the polymorphic C31S substitution in subtype C Tat alters protein stability and LTR-driven transactivation efficiency of this variant relative to clade B Tat [99].

Interestingly, although clade D Tat initially triggered significantly higher peak viral reactivation than the clade C variant, a steeper temporal decline eventually dropped its activity to below clade C levels. Consequently, clades A and D exhibited the most significant disparity in activity loss over time. Our results reveal a cellular restriction of existing Tat activity which varies by clade—particularly between clades A and D—causing differences in Tat’s ability to transactivate latent HIV. The temporal fluctuations in Tat activity observed in the current study may stem from distinct shifts in post-translational modifications mediated by dynamic interactions with chromatin-modifying enzymes. Additionally, variations in protein turnover may play a role; several studies have shown that host E3 ubiquitin ligases can target Tat for polyubiquitination and degradation via proteasomal or autophagy–lysosomal pathways [100,101,102]. Therefore, future research is warranted to investigate the mechanistic basis behind this diminished transactivation capacity, alongside examining the extent to which these Tat–host interactions differ among intra-clade and inter-clade variants.

Because a major fraction of P-TEFb is sequestered by 7SK snRNP in actively dividing cells, its availability for transcriptional control relies on how efficiently it is mobilized from this complex. Immunoprecipitation of FLAG-Tat clade variants and normalization of co-precipitated proteins to the corresponding expression levels of Tat revealed statistically significant differences in the extent of P-TEFb binding and association with the 7SK snRNP core components MEPCE and LARP7. Notably, clades A and C Tat interacted robustly with P-TEFb, significantly outperforming clade D Tat, while exhibiting the lowest association with MEPCE and LARP7. These data suggest that the P-TEFb bound to A and C Tat represents the liberated form, implying these variants reverse the enzyme’s sequestration more efficiently than the other variants examined. Future studies will pinpoint the sequence or structural determinants of Tat underlying this differential release of P-TEFb from 7SK snRNP. While both clades A and C Tat display high normalized P-TEFb binding and low 7SK association, they diverge in their transactivation activity—A Tat drives high peak reactivation with moderate decay, whereas C Tat exhibits significantly lower peak activity. By contrast, D Tat demonstrates moderate P-TEFb and 7SK binding with high initial peak transactivation which is uniquely susceptible to rapid temporal restriction. Since the ability of clade A Tat to efficiently mobilize P-TEFb coincides with its superior transactivation capacity, we propose this variant as the optimal candidate for ectopic Tat utilization in latency-reversal studies.

### 4.3. Utility of the Jurkat 2D10 Model for Comparative Tat Transactivation Analysis

While latently infected CD4^+^ T cells from people living with HIV harbor proviruses across diverse genomic loci—where local chromatin architecture, histone modifications and integration orientation heavily influence baseline transcriptional silencing and responsiveness to LRAs [103,104,105,106,107]—the use of the single-integration Jurkat 2D10 clonal model offers critical methodological advantages for dissecting intrinsic Tat protein functions. First, the clonal nature of the 2D10 system holds the genomic microenvironment and chromatin state constant across all experimental groups, eliminating integration-site variability as a confounding factor. While proviral integration sites in primary cells dictate the absolute threshold of reactivation—with proviruses in deep heterochromatin requiring greater transcriptional activation than those in euchromatic regions—the relative hierarchy of transactivation potency and temporal decay observed among the Tat clades is fundamentally dictated by Tat–intrinsic host factor interactions rather than local epigenetic context. Second, potential confounding effects arising from the 2D10′s H13L Tat variant provirus are minimized or accounted for by the following factors:

- *Uniform genetic background:* Because the single integrated 2D10 provirus is identical across all experimental groups, any downstream contribution or interaction from endogenous *in-cis* H13L Tat represents a uniform baseline. Thus, observed differences in reactivation kinetics reflect intrinsic properties of the ectopically expressed *in-trans* Tat clade variants.
- *Temporal persistence dynamics:* Rather than confounding results, the rapid-shutdown phenotype of H13L Tat—which exhibits accelerated transactivation decay and allows the provirus to rapidly re-establish latency [63]—makes the system particularly sensitive for evaluating how effectively each *in-trans* Tat variant sustains LTR transactivation and is counteracted by cellular restriction events.
- *Low background activation:* While the transactivation-deficient C22G Tat mutant confirms that baseline *in-cis* H13L Tat expression does not drive spontaneous background reactivation in the absence of a functional activator, we note that this control does not exclude a potential contribution from endogenous H13L Tat after functional ectopic Tat has initiated transcription. Nevertheless, because all conditions share this identical background, relative comparisons between the clade variants remain robust.

Finally, the use of a standardized Clade B TAR element in the 2D10 provirus—which contains the universally conserved UCU bulge required for direct Tat binding and the 6-nucleotide apical loop required for Cyclin T1/P-TEFb binding [35]—further strengthens the experimental design. By evaluating all FLAG-Tat variants against an identical TAR target, we eliminated TAR sequence variation as a confounding variable to assess biochemical differences in P-TEFb binding and 7SK snRNP dynamics. We acknowledge, however, that utilizing a single standardized Clade B TAR element and a clonal integration site is a limitation of this study. This design does not establish that the relative ranking of Tat variants will persist across cognate clade-matched TAR sequences or different chromosomal integration sites, which can influence local chromatin environments and transcriptional competency. Although naturally occurring clade variations in TAR can introduce minor sequence changes in the lower stem, the primary binding loops remain functionally conserved, and published cross-transactivation studies demonstrate efficient transactivation across clades [108,109,110]. Thus, the conservation of core bulge and loop domains indicates that relative differences in Tat:P-TEFb assembly and temporal activity loss are robust against TAR sequence context. Nonetheless, testing Tat variants against their cognate, clade-matched TAR sequences and evaluating multiple integration sites will be important avenues for future work to explore potential co-evolutionary fine-tuning.

## Data Availability

The raw data from this study are available from the corresponding author upon request.

## Supplementary Materials

The following supporting information can be downloaded at: https://www.mdpi.com/article/10.3390/v18101137/s1. Supplementary Figure S1. Densitometric quantification of co-precipitated proteins without normalization to corresponding immunoprecipitated Tat. (A) Extent of Tat association with P-TEFb. (B) Extent of Tat association with 7SK snRNP protein components MEPCE and LARP7. Plotted data are from at least three independent IP experiments. Statistical significance was determined by a one-way ANOVA with a post hoc Student’s *t*-test. *p* values from the post hoc *t*-test are presented in Supplementary Table S2. Supplementary Figure S2. Scheme illustrating the timeline of the experiment presented in Figure 7, from transduction with FLAG-Tat constructs and creation of the stable lines by puromycin selection to spontaneous viral reactivation by media replenishment followed by monitoring of the waning trans-activation activity. Supplementary Figure S3. Cell viability minimally changes despite temporal fluctuations in Tat activity. Following a 1:3 media replenishment, viability of Jurkat cells stably expressing FLAG-Tat variants was measured after propidium iodide staining across the time points shown. Plotted cell viability is expressed relative to viability measurements of the Jurkat control. Supplementary Table S1. Key reagents used in the current study. Supplementary Table S2. Stellaris DNA oligonucleotide RNA FISH probes used to detect HIV RNA and 7SK snRNA. Supplementary Table S3. Statistical ANOVA with post hoc Student’s *t*-test analysis for the data shown in Figure 6C,D. Supplementary Table S4. Statistical ANOVA with post hoc Student’s *t*-test analysis for the data shown in Supplementary Figure 1. Supplementary Table S5. Statistical ANOVA with post hoc Student’s *t*-test analysis for the data shown in Figure 7B.
