# Supplementary material for: HIV-1 Tat Forms a Dissociable Complex with 7SK snRNP and Exhibits Clade Differences in Reversing the Sequestration of P-TEFb
Source: Viruses. 2026 Oct 14;18(10):1137. doi: 10.3390/v18101137 (PMC13709444; doi:10.3390/v18101137)
Supplement: Supplementary file 1 [file viruses-18-01137-s001.zip › Supplementary Table S1.pdf]

Supplementary Table S1. Key reagents used in the current study

REAGENT	SOURCE	CATALOG #
**Antibodies**		
Cyclin T1	ThermoFisher	PA5-77892
CDK9	ThermoFisher	MA5-14912
LARP7	ProteinTech	17067-1-AP
MEPCE	ProteinTech	14917-1-AP
Tat (2D1.1)	Custom-synthesized	NT3 2D1.1
Tat (AB69)	Custom-synthesized	AB69
FLAG M2	EMD Millipore	F1804
METTL16	Cell signaling Tech	87538S
HEXIM1	Custom-synthesized	CW194
HA	EMD Millipore	H6908
Anti-rabbit IgG, HRP-linked	Cell signaling Tech	7074S
Anti-mouse IgG, HRP-linked	Cell signaling Tech	7076S
**Other reagents**		
MSCV retroviral expression system	Takara Bio	634401
PhosSTOP	Roche	4906837001
EDTA-free protease inhibitors	Roche	11836170001
Flavopiridol	EMD Millipore	F3055
His-tagged RNAP II CTD	Abcam	ab81888
Anti-FLAG M2 affinity resin	EMD Millipore	A2220
FLAG peptide	EMD Millipore	F3290
Propidium iodide	Cayman Chemical	10008351
Anti-HA agarose beads	Pierce	26181
HA peptide	EMD Millipore	I2149
Memory CD4+ T cell enrichment kit	StemCell Technologies	19157
