# Supplementary material for: HIV-1 Tat Forms a Dissociable Complex with 7SK snRNP and Exhibits Clade Differences in Reversing the Sequestration of P-TEFb
Source: Viruses. 2026 Oct 14;18(10):1137. doi: 10.3390/v18101137 (PMC13709444; doi:10.3390/v18101137)
Supplement: Supplementary file 1 [file viruses-18-01137-s001.zip › Supplementary Table S2.pdf]

Supplementary Table S2. Stellaris DNA oligonucleotide RNA FISH probes used to detect HIV RNA and 7SK snRNA

RNA target	Probe sequences (5' to 3')	
**Total HIV mRNA**	TTCGTTGGGAGTGAATTAGC	CTGCTTATATGCAGGATCTG
	TCCACAGATCAAGGATATCT	ACCCAGTACAGGCAAAAAGC
	AAGTAGCCTTGTGTGTGGTA	CAGATCTGGTCTAACCAGAG
	GGTGTGTAGTTCTGCTAATC	TTCCCTAGTTAGCCAGAGAG
	AGGTCAGTGGATATCTGATC	GCTTTATTGAGGCTTAAGCA
	TACTAGCTTGTAGCACCATC	AGAGTCACACAACAGACGGG
	CTAACTTCTCTGGCTCAACT	GTCTGAGGGATCTCTAGTTA
	TGGTGTTCTCTCCTTTGTTG	GCTAGAGATTTTCCACACTG
	AGGCTCACAGGGTGTAACAA	TGTGTAGTTCTGCCAATCAG
	CAAACCTCCACTCTAACACT	TTCTACCTTATCTGGCTCAA
	TCGGGCCATGTGATGAAATG	ACTTCTCTCTCAGGGTCATC
	TGTAGCAAGCTCGATGTCAG	GCTTATATGCAGCATCTGAG
	AAAGTCCCCAGCGGAAAGTC	TGAGGCTTAAGCAGTGGGTT
	TTGAAGTACTCCGGATGCAG	
**7SK snRNA**	CAGATCGCCCTCACATCC	
	GGGGTGACAGATGTCGCA	
	CAGATCAGCCGAATCAACCCTGGC	
	TTCGGGAGGGACGCACAT	
	GGGATGGTCGTCCTCTTC	
	CTTGACCGAAGACCGGTC	
	GCGCAGCTACTCGTATAC	
	TGGAGGTTCTAGCAGGGG	
	TGGACCTTGAGAGCTTGT	
	TGTCTGGAGTCTTGGAAG	
	AAAGGCAGACTGCCACAT	
